# The Effect of Different Oxygen Surface Functionalization of Carbon Nanotubes on the Electrical Resistivity and Strain Sensing Function of Cement Pastes

**DOI:** 10.3390/nano10040807

**Published:** 2020-04-23

**Authors:** B. Del Moral, I. Martín Gullón, R. Navarro, O. Galao, F.J. Baeza, E. Zornoza, B. Calderón, I. Rodríguez, N. Arnaiz, M.D. Romero Sánchez, P. Garcés

**Affiliations:** 1 Department of Civil Engineering, University of Alicante, Carretera San Vicente, s/n., 03690 Alicante, Spain; beatriz.dmd@ua.es (B.D.M.); rosa.navarro@ua.es (R.N.); oscar.galao@ua.es (O.G.); fj.baeza@ua.es (F.J.B.); emilio.zornoza@ua.es (E.Z.); 2 Applynano Solutions, S.L. Scientific Park of Alicante, Carretera San Vicente, s/n., 03690 Alicante, Spain; gullon@ua.es (I.M.G.); blanca.calderon@applynano.com (B.C.); iluminada@applynano.com (I.R.); noelia.arnaiz@applynano.com (N.A.); md.romero@applynano.com (M.D.R.S.)

**Keywords:** carbon nanotubes, cement, mechanical properties, electrical properties, functionalization, sensing function

## Abstract

Different studies in the literature indicate the effectiveness of CNTs as reinforcing materials in cement–matrix composites due to their high mechanical strength. Nevertheless, their incorporation into cement presents some difficulties due to their tendency to agglomerate, yielding a non-homogeneous dispersion in the paste mix that results in a poor cement–CNTs interaction. This makes the surface modification of the CNTs by introducing functional groups on the surface necessary. In this study, three different treatments for incorporating polar oxygen functional groups onto the surface of carbon nanotubes have been carried out, with the objective of evaluating the influence of the type and oxidation degree on the mechanical and electrical properties and in strain-sensing function of cement pastes containing CNTs. One treatment is in liquid phase (surface oxidation with HNO_3_/H_2_SO_4_), the second is in gas phase (O_3_ treatment at 25 and 160 °C), and a third is a combination of gas-phase O_3_ treatment plus NaOH liquid phase. The electrical conductivity of cement pastes increased with O_3_- and O_3_-NaOH-treated CNTs with respect to non-treated ones. Furthermore, the oxygen functionalization treatments clearly improve the strain sensing performance of the CNT-cement pastes, particularly in terms of the accuracy of the linear correlation between the resistance and the stress, as well as the increase in the gage factor from 28 to 65. Additionally, the incorporation of either non-functionalized or functionalized CNTs did not produce any significant modification of the mechanical properties of CNTs. Therefore, the functionalization of CNTs favours the de-agglomeration of CNTs in the cement matrix and consequently, the electrical conductivity, without affecting the mechanical behaviour.

## 1. Introduction

Concrete is considered the traditional construction material par excellence in the world. Nevertheless, there is a demand for technological advances in response to new innovative requirements [1], based on sustainability issues as well as on safety criteria. Multifunctional concrete composites are a new generation of materials which provide infrastructures with the ability to perform simultaneous new functions themselves, in addition to the usual structural role [2,3]. When materials with these functions produce a signal variation which could be detected by an external device, they are also called smart materials. One key application of smart materials is structural health monitoring (SHM), which is made possible thanks to their intrinsic self-sensing properties, also known as piezoresistive properties [4,5,6,7].

One of the most promising functional admixtures for obtaining self-sensing materials are carbon nanotubes (CNTs), due to their excellent mechanical and electrical properties [8,9,10]. The composite that results from including CNTs into a usual Portland cementitious matrix is able to state mechanical parameters by measuring the electrical properties of the composite, i.e., the piezoresistive behaviour [11,12,13,14,15]. The mechanical parameters include strain (or deformation), stress (or external force), crack and damage under static and dynamic conditions, whereas the detecting electrical parameter consists of a rather simple resistance measurement. This strain-sensing property shown by CNT-cement-based composites immediately leads to an interesting application of reinforced concrete structures: their SHM [10,16,17]. Nevertheless, the incorporation of CNTs to cement presents some difficulties due to their tendency to agglomerate due to its hydrophobic character, yielding a non-homogeneous dispersion through the cement phase which results in a poor cement–CNTs interface [18]. One possible strategy to improve that interaction is the surface modification of the CNTs, by introducing hydrophilic functional groups onto the CNTs’ surface [19,20]. The surface chemistry of CNTs, and carbon-based materials in general, may be modified by different procedures and treatments (chemical, gas, thermal, electrochemical, etc.), mostly with the objective to functionalize the surface of the CNTs with specific chemical groups for different applications [21,22,23]. With these surface treatments, the quantity and type of functional groups can be modified and, consequently, some material properties may be customized, such as reactivity, wettability, etc. The main objective of surface treatments is to increase the compatibility between the CNTs and the matrix, to get a proper dispersion and, in the end, to create strong interactions that make the stress transfer possible [24]. This is particularly important for cement matrices, considering the different chemical nature of CNTs (carbon-based) and the polar chemical nature of the cement paste components.

The first oxygen functionalization treatments were originally reported for carbon fibres to shift from a non-polar to polar character on their surface [25,26]. Typical oxygen incorporation treatments varied from liquid phase HNO_3_/H_2_SO_4_ (aqua regia), for incorporating mainly acidic carboxyl groups [27,28,29,30], to gas phase ozonization, which deals with incorporating base character groups [31,32]. In previous research works, a significant improvement in terms of ductility, and the tensile and flexural strengths of mortars or concretes containing oxidized fibers has been reported [33,34,35]. Moreover, non-mechanical properties are also affected by oxidation treatments, such as the electrical contact resistivity between fibers and cement matrix [36], and dispersion and solubility in water and organic solvents [37,38]. The functionalization technologies have been adapted for the treatment of micro- and nanoparticles, such as CNTs [39].

For these materials, concerning the intensity of the oxygen incorporation over the CNTs’ surface, different authors [40,41] have indicated that the duration of the treatment has significant effect on both the degree of defects and the functionality of the treated CNTs.

K.A. Wepasnick et al. [42] indicated that different oxidizing conditions are likely to affect both the concentration of oxygen atoms incorporated into the CNTs and the distribution of oxygen-containing functional groups. Moreover, oxygen-containing groups are also sensitive to the identity of the oxidant, as demonstrated by the authors in this work.

These oxidation treatments may even produce clear surface damage over CNTs, leading to the formation of debris, which can be washed away simply through NaOH treatment, as reported by Verdejo et al. [43]. Additionally, Konsta-Gdoutos et al. [12] compared the electrical resistivity of cement composites reinforced with well dispersed carbon nanofibers with those composites with pristine carbon nanofibers, obtaining a good correlation between the electrical resistivity and the dispersion degree of the nanomaterial in the matrix. Particularly, on CNT-cement composite pastes, Jianlin et al. [44] reported that, with moderate functionalization using a photo-assisted Fenton, CNTs were found to easily disperse in an aqueous system aided with low fraction of dispersants, whose inclusion is typically used to improve the mixing of the CNTs. They used Fenton-functionalized CNTs to reinforce both the mechanical and electromechanical properties of cementitious composites for developing intrinsic self-sensing sensors. No work has been found in the literature referring to different types of oxidation-functionalization treatment with the aim of improving the sensing function of small conductive-cement-based sensors by the addition of CNT. In this study, different oxidation treatments based on the use of H_2_SO_4_-HNO_3_, O_3_ and O_3_ followed by NaOH (O_3_-NaOH) have been carried out to promote the functionalization of the CNTs’ surface, with the main goal of the evaluation of the influence of the type of treatment and the achieved oxidation degree on the mechanical and electrical properties, and on the strain-sensing function of the cement paste.

## 2. Materials and Methods

### 2.1. Materials and Specimen Fabrication

Prismatic cement paste specimens of standard size, 4 × 4 × 16 cm^3^ dimension, were fabricated according to the European standard EN 196-3:2005. The materials included in the preparation consisted of Portland cement type EN 197-1 CEM I 52.5R, supplied by Cemex España S.A., (Madrid, Spain), CCVD (Catalytic Chemical Vapor Deposition) commercial multiwall CNTs Graphistrenght C100, manufactured and supplied by Arkema (Colombes, France), whose main characteristics are shown in Table 1, superplasticizer Sika viscocrete 20-HE, supplied by SIKA (Madrid, Spain) and demineralized water.

### 2.2. Oxygen Funcionalization Treatments

Different procedures were carried out for the oxygen surface functionalization of CNTs, based on the oxidant attack with HNO_3_-H_2_SO_4_-and an O_3_ atmosphere, the latter combined with an NaOH wash treatment. Previous optimization work was carried out by the authors evaluating each different concentrations and exposure times, flow rates, temperature, etc., for each treatment. Optimized experimental conditions for each treatment were selected and carried out prior to the incorporation of CNTs to the cement paste. The chemical and morphological modifications produced on the surface of CNTs because of the treatments were evaluated by different experimental techniques and compared with the original CNTs. Infrared Spectroscopy (IR) using a JASCO IRT-5200 (Madrid, Spain) with an ATR mode has been used for the identification of chemical bonds introduced on the CNTs’ surface by the oxidation treatments. Surface composition has been quantified by using an X-ray Photoelectron Spectrometer (XPS) K-Alpha of Thermo-Scientific (Madrid, Spain), and the morphology of the differently treated CNTs has been examined by Transmission Electron Microscopy (TEM) using a JEOL JEM-1400 Plus (Freising, Germany). After the oxygen functionalization, the following treatments were carried out:

#### 2.2.1. CNTs Surface Treatment with H_2_SO_4_-HNO_3_

The solution to attack the CNTs surface consisted of one part of concentrated HNO_3_ (68%, GPR Rectapur) per three parts of concentrated H_2_SO_4_ (95%, Analar Normapur) in volume. A quantity of 45 g of CNTs was immersed and magnetically stirred in 1.5 l of the acid mixture for 3 h at 80 °C under reflux. After that, the solid was filtered and washed with demineralized water until reaching a neutral pH. Finally, the solid part was dried for 1 day in an oven at 70 °C.

#### 2.2.2. CNTs Surface Treatment with Ozone (O_3_)

A quantity of 1 g CNTs were subjected to an O_3_ atmosphere for different times (30 and 60 min) in a rotating reactor using an air flow of 0.5 L/min and 95% intensity. The surface modification was carried out at room temperature and at 160 °C.

#### 2.2.3. CNTs Surface Treatment with O_3_-NaOH

Firstly, the CNTs were subjected to the O_3_ treatment for 30 min. Subsequently, the CNTs were further treated with 10 M NaOH solution for 24 h under mechanical stirring. The modified CNTs were washed until reaching a neutral pH and dried for 1 day at 70 °C.

### 2.3. Dispersion Procedure of CNTs in Cement Pastes

Water-based dispersions of CNTs (different wt% respect to cement) were prepared by using a high shear mixer for 10 min. After that, superplasticizer Sika Viscocrete 20-HE was added. Then, a 10 min ultrasound treatment was applied to the dispersions using an ice bath to avoid an excessive temperate increase in the mix.

Subsequently, dispersions of original and treated CNTs were incorporated with cement by using a planetary mixer. Then, dispersions were poured into the mixer container and cement was added in three parts of 600 g. Each part was mixed for 2 min at low speed. Finally, when all cement parts had been added, the paste was mixed for 1 min at high speed. A water/cement ratio of 0.5 was used in all cases.

Cement pastes were poured in moulds and compacted with a vibrating table. After 24 h in humid chamber, the samples were extracted from the moulds. All samples were cured at 100% of relative humidity (RH) and 20 °C for 28 days, according to UNE EN-80-101-88. After 28 days of curing time, the specimens were kept at laboratory conditions before their characterization.

### 2.4. Mechanical Tests

**Figure 1:**
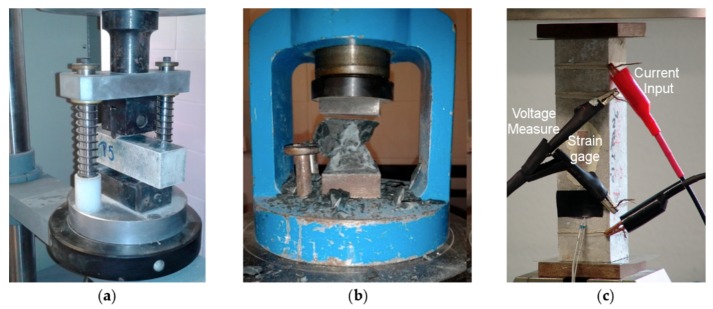
Experimental setup for mechanical strength tests: (**a**) Bending strength; (**b**) Compressive strength; and (**c**) electrical resistivity measures and strain sensing tests.

Mechanical tests were performed under laboratory conditions according to European Standard EN 196-1. Flexural and compressive strength tests on prismatic specimens were conducted according to European standard EN 196-1:2005, with an ME-402/20 mechanical testing machine (Servosis, S.A., Madrid, Spain). For the flexural strength test (Figure 1a), prismatic specimens with dimensions of 4 × 4 × 16 cm^3^ were used. This test consists of the application of a three-point bending stress at a loading rate of 50 ± 10 N/s up to the break of the specimen. The two resulting pieces of the broken specimen were subjected to compressive strength tests (Figure 1b), in which a uniaxial compressive loading at a rate of 2400 ± 200 N/s was applied to the sample up to the break.

### 2.5. Electrical Resistivity Tests

After curing, specimens were externally dried and electrically conductive silver paint (Pelco Conductive Silver 187) was applied around the perimeter of four cross-sections planes (Figure 1c) which were parallel to the end surfaces. Four copper wires were wrapped around each silver painted perimeter in order to form four electrical contacts, as needed for the four-probe method. This method consists of applying a fixed electrical current with an AC/DC current source (Keithley Model 6220, Beaverton, OR, USA) at the outer electrodes, while the voltage was measured between the inner electrodes using a digital multimetre (Keithley Model 2002, Beaverton, OR, USA). Hence, electrical resistance may be calculated applying Ohm’s law.

### 2.6. Self-Sensing Tests

Several consecutive loading-unloading cycles of compressive stress were applied to each specimen, while a fixed current of 1 mA was applied to the external electrodes (Figure 1c). Specimens were first loaded up to 1.5 kN and then five or six cycles were applied up to 6.5 kN (4 MPa) and 13.5 kN (8.4 MPa), with a loading rate of 200 N/s.

## 3. Results and Discussion

### 3.1. Characterization of CNTs Treated with H_2_SO_4_-HNO_3_, O_3_ and O_3_-NaOH

**Figure 2:**
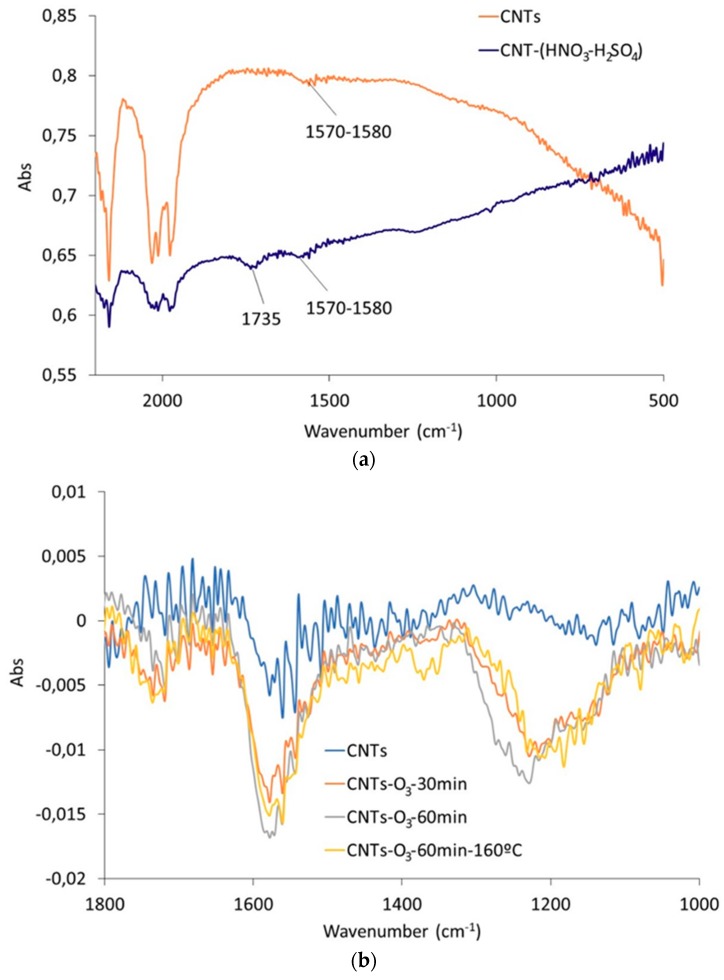

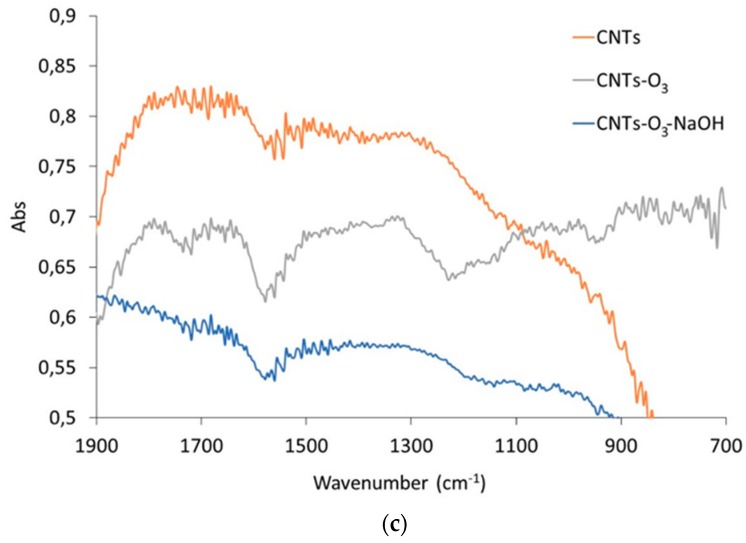
IR spectra of (**a**) original CNTs and CNTs treated with HNO_3_-H_2_SO_4_; (**b**) CNTs treated with O_3_ for different times and temperature and (**c**) CNTs treated with O_3_-NaOH.

CNTs treated with H_2_SO_4_-HNO_3_ and CNTs treated with O_3_ and O_3_-NaOH were characterized by IR spectroscopy to identify chemical groups on the CNTs surface (Figure 2a–c). Both H_2_SO_4_-HNO_3_ and O_3_ treatments produced the formation of polar groups containing oxygen on the CNTs’ surface [45]. Figure 2a includes the IR spectra (amplified scale from 500–2200 cm^−1^) of the original CNTs and those treated with HNO_3_-H_2_SO_4_. The main difference in the IR spectra in Figure 2a is the presence of a band at 1735 cm^−1^ identified in the IR spectrum of the CNTs treated with HNO_3_-H_2_SO_4_, which may be ascribed to C=O groups, due to the oxidation of the CNTs’ surface. This band is not observed in the IR spectrum of the original CNTs. On the other hand, Figure 2b includes the IR spectra corresponding to the CNTs treated with O_3_ for 30 and 60 min at room temperature and for 60 min at 160 °C. The amplified scale in the range 1100–1800 cm^−1^ can identify the oxygen-containing groups introduced as a consequence of the O_3_ treatment and not present in the original CNTs, mainly with the presence of the bands at 1200–1230 cm^−1^ ascribed to C-O-C stretching and/or arC-OH, and a lower relative intensity band at 1720–1730 cm^−1^ due to C=O stretching bonds. The broad band with a main peak centered at 1550–1580 cm^−1^ may be ascribed to C=C stretching bonds, typical of the CNTs’ backbone. Comparison of the relative intensity of the bands indicates that there are no significant differences among the O_3_ treatments, only showing a slight increase in the intensity of the band at 1550–1580 cm^−1^ for the CNTs treated for 60 min. The O_3_ treatment carried out at 160 °C does not produce an increase in the oxidation degree compared to the modifications produced by the O_3_ treatment at the same time (60 min) at room temperature. These bands indicate that the oxidation produced by the O_3_ treatment may be predominantly due to the introduction of phenolic and ether groups on the CNTs surface. However, the liquid acid treatment on CNTs mostly leads to the incorporation of C=O groups. No further differences could be determined due to low signal intensity obtained by IR spectroscopy for CNTs and its qualitative nature, which does not allow a precise data interpretation.

Figure 2c includes the IR spectra (amplified scale 700–1900 cm^−1^) of original CNTs and CNTs treated with O_3_-30 min and treated with O_3_ followed by NaOH. The analysis of these spectra indicates that the O_3_ treatment of CNTs produces the formation of C=C and C-O-C bonds, respectively, at 1580 and 1220 cm^−1^, whilst the subsequent NaOH treatment acts as a washing treatment, removing these moieties from the CNTs’ surface (very low relative intensity of the bands at 1580, and mostly at 1220 cm^−1^), leading to a surface composition very similar to the original CNTs. This effect produced by washing with NaOH has been previously observed by Verdejo et al. [43].

Table 2 shows the carbon and oxygen content for the CNTs for the different surface treatments determined by XPS (at%). It can be observed that the different treatments introduce oxygen onto the CNTs surface, with the highest oxygen at% obtained when using a mixture of HNO_3_-H_2_SO_4_ (C/O = 5.8). Besides, the O_3_ treatment also produces oxidation of the CNTs, which is more significant when increasing the treatment time (C/O = 19.7 and 14.9, for the O_3_ treatment during 30 and 60 min, respectively). The O_3_ treatment of CNTs at 160 °C produces a decrease in the oxidation degree compared to the CNTs treated with O_3_ at room temperature. These results agree with those obtained by IR spectroscopy (Figure 2b). The O_3_-NaOH treatment decreased the O content of the O_3_ treatment from 4.8 to 3.9 at%, corroborating the results obtained with IR spectroscopy, but 1.0 at% of Na^+^ was additionally incorporated onto the CNTs’ surface, which may increase the interaction with the inorganic chemical nature of cement.

**Figure 3:**
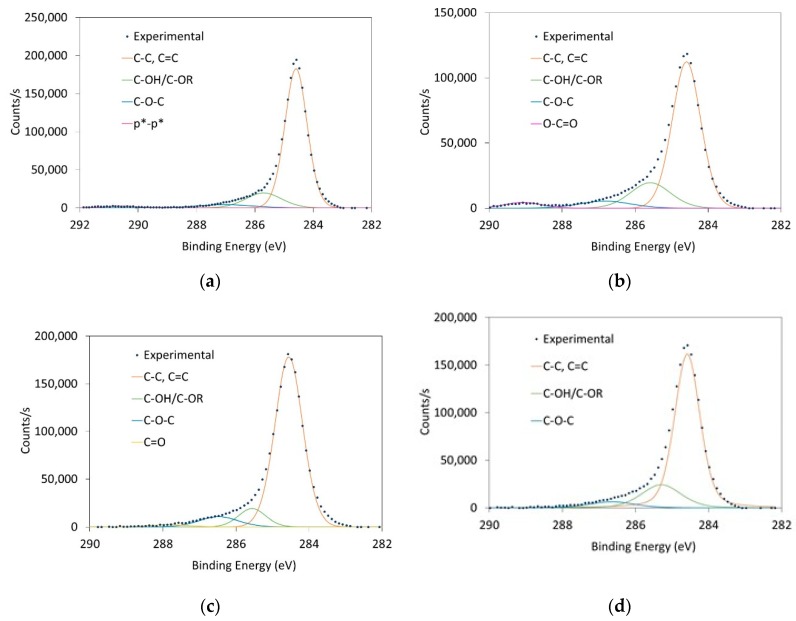
C1s peak deconvolution of (**a**) original CNTs; (**b**) CNTs treated with HNO_3_-H_2_SO_4_; (**c**) CNTs treated with O_3_-30 min and (**d**) CNTs treated with O_3_-NaOH.

The C1s high resolution XPS peaks for the original CNTs, and those treated with HNO_3_-H_2_SO_4_, O_3_ (30 min) and O_3_-NaOH were deconvoluted (Figure 3). The results obtained for the different peaks have been included in Table 3.

The peak at 284.5 eV ascribed to sp2 C-C is, as expected, dominant in all the samples, although its proportion is lower for the HNO_3_-H_2_SO_4_ sample, indicative of a higher oxidation degree with the liquid phase acid treatment. The 285.5 eV peak deconvolution, associated with sp3 C-OH and sp3 C-sp3 C, is also present in all the samples. However, the O_3_ (30 min) introduces a higher proportion of both C-O-C and C=O, 7.1 and 1.9%, whereas HNO_3_-H_2_SO_4_ is the only sample with carboxyl groups (288.5 eV, 3.7%). In all cases, the sp2 carbon hybridization in the original CNTs has been converted into sp3 hybridization after treatments. Moreover, and according to the results observed in the IR spectra, the C=C bonds% increase for the O_3_-treated CNTs, with respect to the original CNTs (82.2 vs. 79%, respectively). This can be explained due to the lower reactivity of O_3_ towards C=C groups. The treatment of NaOH subsequent to the O_3_ process produces the washing of the O_3_-treated CNTs, yielding a surface with a similar composition to the pristine CNTs surface, although it does not present the aromatic π *-π * peak, which is absent in all the differently treated CNTs. This peak is only present in the original CNTs, confirming the surface modification of CNTs by the different treatments (either with the introduction of C-O-C or of more oxidized groups, i.e., carboxylic groups). It is also interesting to mention that when more oxidative treatments (HNO_3_-H_2_SO_4_) are applied, higher COOH concentrations are observed for the CNTs, while the weaker oxidant treatment (i.e., O_3_) introduces the higher C-OH and C=O concentrations.

**Figure 4:**
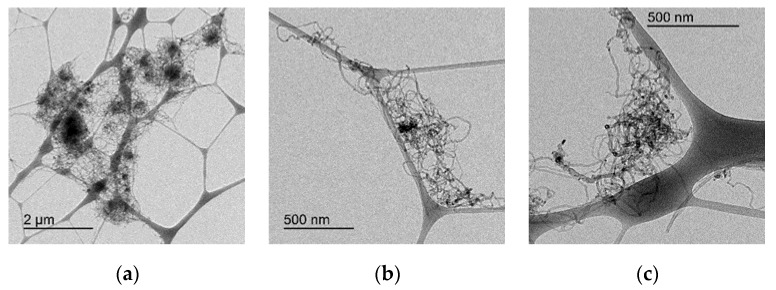
(**a**)–(**c**): TEM micrographs of original CNTs at different magnification scale.

**Figure 5:**
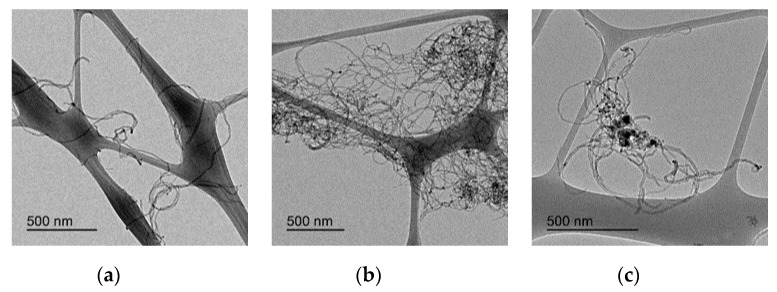
TEM micrographs of: (**a**) CNTs treated with HNO_3_-H_2_SO_4_; (**b**) CNTs treated with O_3_-30 min and (**c**) CNTs treated with O_3_-NaOH.

Additionally to these chemical modifications, some differences in the agglomeration degree of the CNTs due to the different functionalization treatments can be observed by a comparison of the micrographs obtained by TEM in Figure 4 and Figure 5a–c. Figure 4a–c includes the micrographs of the original CNTs (at different magnifications) and Figure 5 the micrographs of the CNTs treated with HNO_3_-H_2_SO_4_ (a), O_3_ (30 min) (b) and O_3_-NaOH (c). The treatment with HNO_3_-H_2_SO_4_ seems to de-agglomerate the original CNTs, producing a mechanical degradation in the CNTs, as they are considerably shorter. Therefore, the aspect ratio of CNT treated with HNO_3_-H_2_SO_4_ is lower than the pristine or O_3_-treated CNTs. The O_3_ and O_3_-NaOH treatments seem to decrease the entanglement degree of the CNTs. This reduction in the CNTs’ entanglement is expected to improve the CNTs’ dispersion in the cement matrix, independently of the oxygen functionalization degree [42,46].

### 3.2. Electrical and Mechanical Properties of Cement Paste with Different Treated CNTs

Table 4 shows the electrical resistivity and compressive strength of cement pastes with functionalized and non-functionalized CNTs cured for 28 days at 100% RH, and the resistivity at 90 days. In the period from 28 to 90 days, the specimens were kept at 60% RH. After 28 days of curing, the cement matrix with the differently treated CNTs shows a slight decrease in the electrical resistivity levels compared to the cement matrix without CNTs. However, after curing for 90 days, the electrical resistivity of the cement matrix with 1% of original CNTs decreases 28%. Additionally, the resistivity of the cement matrices with 1% functionalized CNTs offered a decrease of 68%, 48% and 88% for O_3_, HNO_3_-H_2_SO_4_ and O_3_-NaOH treatments, respectively. Consequently, it can be deduced that the application of the different functionalization treatments improved the CNTs’ dispersion and wettability in the water-based cement matrix, since more homogeneous materials are achieved. With respect to the efficiency of each particular oxidation treatment, the most noticeable decrease in the resistivity was obtained for the attack with O_3_-NaOH.

With respect to the influence of CNTs and the oxidation treatment in the compressive strength, it can be observed that the poor dispersion of the original CNTs decreases the mechanical strength of the composite. Then, if an oxidation treatment is applied, the compressive strength recovers the original value shown by the cement paste without CNTs. That fact would support the improvement in the CNTs’ dispersion achieved by any of the applied treatments, since a low dispersion of the CNTs would offer a matrix with weak points in those zones where the CNTs are agglomerated. Besides, no significant influence of the type of the oxidation treatment is observed in the compressive strength.

### 3.3. Strain Self-Sensing Tests

The main objective of this work is the development of conductive multifunctional cement-based materials for specific applications in the area of civil engineering and architecture. Particularly, interesting smart structural materials are those exhibiting strain-sensing properties, that is, the ability to provide an electrical output that is correlated to their state of strain, also known as a piezoresistive property [10,13,16,17,23,25]. The material’s strain-sensing capacity is defined as the response on the volumetric electrical resistivity (proportional and reversible) due to its strain state [35,36]. The sensing function is based on changes in electrical resistance in conductive-cement-based materials when some stress is applied to it. It is quantified by the gage factor (k) of the conductive element. This function is defined as the ratio of fractional change in resistance divided by the fractional change in deformation, according to Equation (1)

(1) $$k=\frac{\Delta R/R_{0}}{\Delta l/l_{0}}=\frac{\Delta R/R_{0}}{\varepsilon }$$

where Δ*R* is change in electrical resistance; *R_0_* is initial electrical resistance; Δ*l* is the specimen’s deformation; *l_0_* is the initial length of the specimen; and *ε* is the strain.

If a longitudinal increasing compressive stress is applied, the electrical resistance in that direction is reduced because the contact between CNTs themselves and between CNTs and cement paste would become closer and closer. Consequently, the contact electrical resistance would decrease and vice versa. When the stress is removed, the material recovers the initial loading state and the resistivity also recovers the initial value. Both effects are reversible in the material’s elastic range. In the present case, the load regime is elastic, because the applied stress is rather low and the resistivity of the specimens is reversible upon the loading cycle. Considering that the changes in dimensions (length or sections) are not significant, then the changes in resistance must be due to the resistivity changes in the material. That is a material intrinsic property.

**Figure 6:**
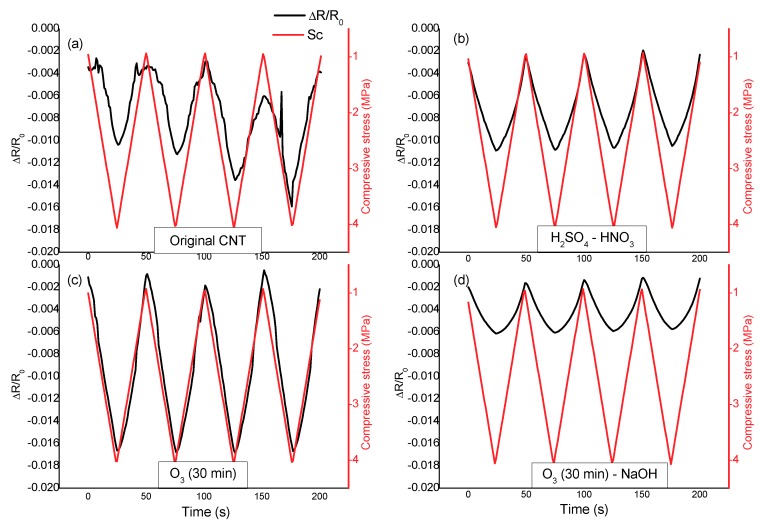
Self-sensing tests for 1.0% of CNT cement paste subjected to different functionalization treatments: (**a**) original CNTs; (**b**) CNTs-(HNO_3_-H_2_SO_4_); (**c**) CNTs-O_3_-30 min; (**d**) CNTs-O_3_-30 min-NaOH. 180 day-old specimens, with a 1 mA DC current, 200 N/s load rate, initial load of 1.5 kN and a maximum load of 6.5 kN.

Figure 6 shows the results for strain-sensing tests for 1.0% of CNTs cement paste subjected to different functionalization treatments, for 180 day-old specimens, with a 1 mA DC current, 200 N/s load rate, a pre-load of 1.5 kN and a maximum load of 6.5 kN. Both stress and fractional changes in electrical resistance data versus time are monitored. From the point of view of the sensitivity, and according to the values of gage factor (k) shown in Table 5, the treatment with ozone improves the level offered by the raw CNTs, even more in the case of the combination of ozone with sodium hydroxide. On the other hand, the acid treatment produced a decrease in the gage factor. However, it has to be pointed out that all the calculated gage factors allow the successful use of these pastes to the sensing function. In order to highlight this statement, it should be taken into account that the typical gage factor for a commercial strain monitoring gage is around 2. Therefore, the sensitivity of any of the proposed CNT-based pastes is higher than the one offered for the classical commercial solution for strain sensing in concrete elements.

However, the most significant parameter to validate the strain-sensing ability of the material is its performance in terms of the quality of the information extracted from the electrical resistance measurement. In the present case, the quality of the correlation between the electrical response and the stress is higher for the materials prepared with the functionalized CNTs than for the original CNT pastes, as can be observed from the Pearson’s correlation coefficient shown in Table 5. Therefore, controlling the resistance of the specimen, the deformations can be monitored. With original CNT cement pastes, although there is a certain correlation, it does not match the performance of the functionalized CNTs for strain-sensing function.

**Figure 7:**
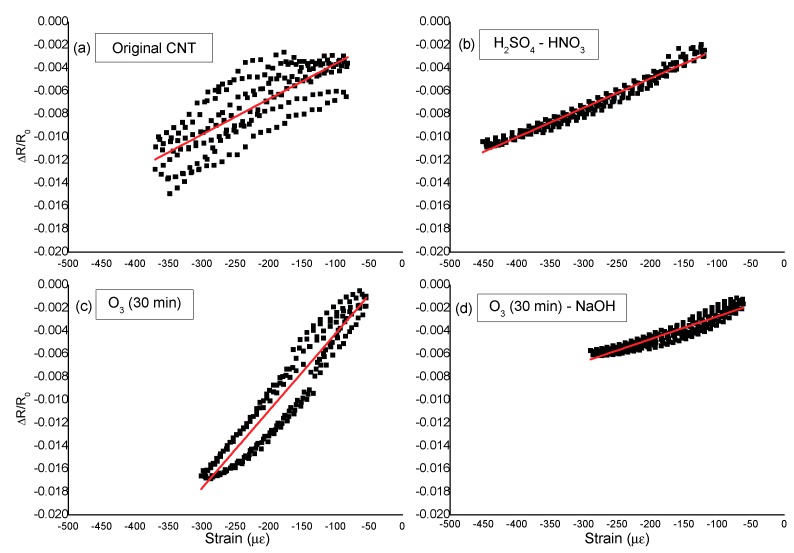
Correlation between the resistance fractional change and the strain for cement pastes with 1% of CNT subjected to different oxidation treatments: (**a**) original CNT; (**b**) CNT-HNO_3_-H_2_SO_4_); (**c**) CNT-O_3_ (30 min); (**d**) O_3_ (30 min)-NaOH. 180 day-old specimens, with a 1mA DC current, 200 N/s load rate, initial load of 1.5 kN and a maximum load of 6.5 kN.

The importance of the R^2^ coefficient in the ability of the CNT-cement paste to monitor the strain can be clearly observed in Figure 7, in which the resistance fractional change vs. the strain is plotted for cement pastes with CNT subjected to different oxidation treatments.

The following hypothesis can be used to explain the improvement in the quality of the strain-sensing behaviour observed for cement pastes with functionalized CNTs: according with the increase in the polarity of the CNTs surface produced by the different functionalization treatments, an improvement in the dispersion easiness of the CNTs in the cement matrix should be expected because the de-agglomeration of the CNTs would be favoured by the creation of oxygen-containing groups, which improves the wettability of the CNTs. This fact would explain the better behaviour shown by the functionalized CNTs in the strain-sensing tests. The general decrease in the resistivity of the pastes with treated CNTs cannot be explained by the chemical changes in the surface of the CNTs, since the opposite result would be expected because the treatment would reduce the resistivity of the CNTs. Therefore, this decrease in the resistivity of the paste with the treated CNTs can be explained by the improvement in the dispersion of the CNTs. The better dispersion would produce a more homogeneous paste that would offer a better strain-sensing response. This argument is also supported by the decrease in the compressive strength that was observed for the non-treated CNT-cement paste, because a poor CNT dispersion creates low-density agglomerates consisting of CNT skeins with air entrapped inside, which would reduce the strength of the composite.

The findings of the present work should encourage the importance of seriously considering the dispersion of CNTs in a cement matrix. As was previously commented, different strategies could be adopted for promoting the best dispersion of CNT, such as the use of chemical dispersants and the application of sonication methods, but also the surface treatments of the CNT could be used to this purpose. Otherwise, the high cost devoted to incorporating such exclusive and expensive admixtures for the development of new features of concrete (strain-sensing, heating, EMI shielding and others) would be wasted.

## 4. Conclusions

The following conclusions summarize the most significant findings of the present study:

1. The electrical conductivity of cement pastes increased with the incorporation of CNTs functionalized with HNO_3_-H_2_SO_4_ and mainly with the O_3_ and O_3_-NaOH treatments;
2. The incorporation of functionalized CNTs did not produce any significant modification of the mechanical properties of cement paste. On the other hand, the presence of the non-treated CNTs negatively affected the compressive strength of the cement paste, indicating a poor dispersion of CNTs in the cement paste matrix;
3. The functionalization of the CNTs has clearly improved the strain-sensing performance of the CNT-cement pastes, particularly in terms of the accuracy of the correlation between the resistance and the stress. This improvement is associated with the better dispersion achieved by the oxidized CNTs compared to the non-functionalized CNTs;
4. This manuscript demonstrates the feasibility of using functionalized CNTs as self-sensing materials for cement pastes, increasing the potential applications of CNTs for structural health monitoring purposes.

## Figures and Tables

**Table 1:** Main properties of carbon nanotubes (CNTs).

Description	CCVD Multi-Wall Carbon Nanotubes	
Appearance	Black powder	
Powder characteristics	Apparent density	50–150 kg/m^3^
	Mean agglomerate size	200–500 μm
	Weight loss at 105 °C	< 1%
CNTs characteristics	C content	> 90 wt%
	Free amorphous carbon	Not detectable (SEM/TEM)
	Mean number of walls	5–15
	Outer mean diameter	10–15 nm
	Length	0.1–10 μm

**Table 2:** Carbon and oxygen content (at%) of CNTs treated with HNO_3_-H_2_SO_4_, O_3_ and O_3_-NaOH.

Treatment	C/O	C (at%)	O (at%)
None	100	99.1	0.9
HNO_3_-H_2_SO_4_	5.8	85.4	14.6
O_3_ (30 min)	19.7	95.2	4.8
O_3_ (60 min)	14.9	93.7	6.3
O_3_ (60 min-160 °C)	21.3	95.5	4.5
O_3_ (30 min)-NaOH	24.3	95.0	3.9

**Table 3:** Bonds composition (%) of CNTs treated with HNO_3_-H_2_SO_4_, O_3_ and O_3_-NaOH.

Binding Energy (eV)	Chemical Bonds	CNT (Bonds%)	CNT-HNO_3_-H_2_SO_4_ (Bonds%)	CNT-O_3_ (30 min) (Bonds%)	CNT-O_3_-NaOH (Bonds%)
284.5	C=C, C-C	79	72.1	82.2	76
285.3–285.6	C-OH/C-OR	14	17.7	8.8	18
286.4–286.8	C-O-C	5	6.5	7.1	6
287.8	C=O			1.9	
289.0	O-C=O		3.7		
290.6	π *-π *	2			

**Table 4:** The resistivity and mechanical properties of cement pastes with functionalized and non-functionalized CNTs cured for 28 days at 100% RH, and then until 90 days at 60% RH.

CNT (%)-Treatment	Resistivity (Ω·cm) 28 Days	Resistivity (Ω·cm) 90 Days	Compressive Strength (MPa) 28 Days
0%-N/A	1716	40,107	51.0
1%-None	1513	29,103	45.0
1%-H_2_SO_4_-HNO_3_	1291	20,747	53.0
1%-O_3_ (30 min)	1414	12,696	51.5
1%-O_3_ (30 min)-NaOH	1473	5035	52.3

**Table 5:** Gage factors (k) and Pearson’s correlation coefficients in the sensing function of CNTs pastes with different oxidation treatments.

	Original	HNO_3_-H_2_SO_4_	O_3_ (30 min)	O_3_ (30 min)-NaOH
k	30.8	25.6	67.2	19.7
R^2^	0.64	0.97	0.90	0.89
